# Development of Functional Dairy Byproducts-based Beverages Using Rutab Date and Probiotic *Apilactobacillus Kunkeei* EABW06

**DOI:** 10.1007/s12602-025-10599-y

**Published:** 2025-05-29

**Authors:** Hany Elkashef, Awad A. Awad, Ashwak Abdel Moneim Hassan

**Affiliations:** Dairy Department, Faculty, of Agriculture, Cairo University, PO Box 12613, Giza, Egypt

**Keywords:** Beverages, Dairy byproducts, Fermentation, Probiotic, Rutab date

## Abstract

This study was designed to valorize dairy byproducts including cheese whey and buttermilk through developing fermented beverages using a novel isolated *Apilactobacillus kunkeei* EABW06 strain from Egyptian bee’s wax. Cheese whey or buttermilk was fermented with *A. kunkeei* and compared to fermented whey or buttermilk supplemented with Rutab date pulp on day one or after 15 days of cold storage. Physicochemical, microbiological, sensory, proteolytic, and various functional properties were investigated. Fermented buttermilk beverages particularly supplemented with date pulp had the highest viscosity and water-holding capacity at the beginning or after 15 days of cold storage. The supplementation with date pulp led to increase the viable count of *A. kunkeei*. Compared to fermented whey beverages, fermented buttermilk beverages recorded the greatest scores of sensory attributes. The proteolytic, ACE-I, and antioxidant activity enhanced in fermented buttermilk supplemented with or without date pulp. A storage period exhibited a positive effect on the proteolysis, ACE-I, and DPPH radical scavenging properties of fermented beverages. All fermented beverages showed a great inhibitory impact against the growth of various species of fungi and bacteria. Fermented buttermilk beverages demonstrated the highest cytotoxicity against Caco2 cell lines with IC_50_ values of 81.22–86.89 μg/mL. However, fermented whey beverages had the strongest inhibition of α-amylase and α-glucosidase enzymes. These findings propose that whey or buttermilk serves as an effective medium for the growth of *A. kunkeei* and potentially enabling the development of innovative fermented dairy beverages with beneficial health effects.

## Introduction

Globally, the issue of food waste and byproducts is worsening due to increasing environmental pollution, rising processing costs, and their direct correlation with the malnutrition and starving issue. Consequently, one of the sustainable development goals is to reduce hunger through valorization of food waste or byproducts by converting them into value-added products [1]. The releasing of dairy byproducts has increased in parallel with the development of the dairy industry, demanding the requirement for their sustainable valorization. Skim milk, cheese whey, and buttermilk are considered the main byproducts of dairy industry, which are rich in inorganic and organic compounds, rendering them potential contributors to environmental pollution [2].

During cheese manufacturing, whey is produced and is known as an aqueous enriched with nutrients and bioactive molecules. Whey is generated in quantities about 80–90% related to those of the milk used during cheese making and consequently demands suitable direction [3]. Cheese whey is composed of lactose, protein such as β-lactoglobulin, α-lactalbumin, bovine serum albumin, lactoferrin, essential amino acids, and other bioactive components. It possesses a great polluting possibility because of its high chemical O_2_ demand (50–80 g/L) and elevated biochemical O_2_ demand (40–60 g/L) that are due to its high content of lactose [4, 5]. Whey powder, whey protein concentrate, isolate, and other whey byproducts are generated by different techniques and are used to manufacture or prepare value-added products including functional beverages [6, 7]. Buttermilk is an aqueous byproduct obtained during butter manufacturing and is considered a functional dairy food because it contains bioactive components derived from milk fat globule membrane (MFGM). Buttermilk is identical to whey in lactose and ash content but comprises a greater quantity of proteins [8, 9]. The health benefits of buttermilk are attributed to containing bioactive components such as mucin-like glycoproteins, butyrophilin, adipophilin, breast cancer type 1 and 2 sensitivity proteins, phospholipids and sphingolipids [10, 11].

Several technological approaches have been discussed to produce functional food products based on dairy byproducts. In this regard, development of fermented whey- or buttermilk-based beverages seems to be the exceedingly easiest and economical solution for the application of dairy byproducts in human nourishment [9]. Whey and buttermilk have been confirmed to be an excellent environment for the growth of lactic acid bacteria (LAB) and probiotic microorganisms where these byproducts are a superb source of nutrients and lactose that can promote the growth of fermentative microorganisms. Development of whey and buttermilk beverages with high nutritional and functional characteristics is closely associated with using novel LAB strains [12]. Given the above-mentioned, this study was designed to develop novel fermented whey- and buttermilk-based beverages using *Apilactobacillus kunkeei*, a fructophilic lactic acid bacterium isolated from Egyptian beeswax that acts on fructose as a carbon source, and several studies have been confirmed its probiotic characteristics [13, 14]. Hence, Rutab date pulp was used to investigate its effect on the growth of *A. kunkeei* in the novel fermented beverages. Physico-chemical, microbiological, sensory, and functional characteristics were examined in novel fermented beverages.

## Materials and Methods

### Materials

Fresh sweet whey and buttermilk were produced from manufacturing rennet cheese and cream churning. Whey protein concentrate (53%) and buttermilk powder were procured from CP Kelco, Huber Company (Georgia, USA) and Bob’s Red Mill Natural Foods Company (Milwaukie, USA), respectively. Lacta 533 stabilizer (modified starch E1422, pectin 440, mono and diglycerides E471) was obtained from Misr Food Additives (MIFAD) Company (Cairo, Egypt). All chemicals were purchased from Sigma-Aldrich (Egyptian Int. Center for Imports, Cairo, Egypt).

Rutab date fruit was purchased from local Egyptian market. For preparation of date pulp, Rutab dates were washed, peeled off, destoned, and the obtained pulp was mashed, filled into PE-bags, and kept at −20 ± 2 °C until utilized.

The strain of *Apilactobacillus kunkeei* EABW06 was previously isolated from honeybee bee’s wax and identified (under publication). To activate *Apilactobacillus* culture, 0.1 mL of stock culture was inoculated in 10 mL of de Man Rogosa Sharpe (MRS) broth medium containing 2% fructose sugar and incubated for 24 h at 30 °C to viable counts approximately log 8 CFU/mL. Activated starter culture was inoculated in sterilized skim milk (1%, w/v) and reactivated at 30 °C until coagulation.

### Preparation of Beverages

**Fig. 1:**
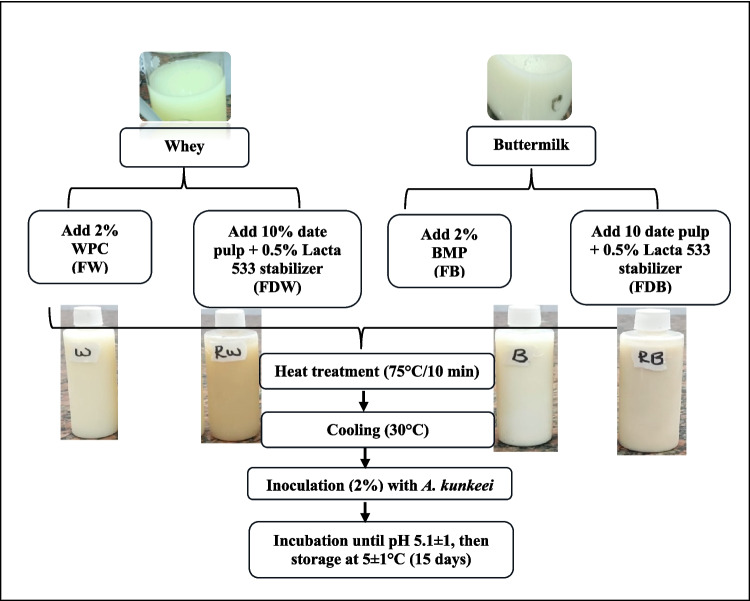
Flowchart for the preparation of fermented beverages. WPC: whey protein concentrate; BMP: buttermilk powder

Four beverages formulation were prepared from whey and buttermilk at laboratory scale as illustrated in Fig. 1. The probiotic *Apilactobacillus kunkeei* culture was added to each fermented beverage at ratio 2% with the initial inoculum count Log 7 CFU/mL. All fermented beverages were three replicated.

### Water-soluble Extract (WSE) Preparation

Two milliliters of fermented beverage were blended well with one mL of distilled water and 5 mL of trichloroacetic acid solution (TCA, 12%). Ten min post-standing, the mixture was centrifugated for 30 min at 10,000 x*g*, and the supernatant (WSEs) were separated, collected and stored at −40°C until analysis [15].

### Chemical Composition and PH

Total solids, protein, fat, and ash of fermented beverages were estimated according to the methods of AOAC [16]. The values of pH were measured by a digital pH meter (Adwa AD11, Adwa Instruments’, Szeged, Hungary). The percentages of titratable acidity were evaluated using titration by sodium hydroxide (0.1 N) and were calculated using the following equation:

$$\%\ \text{Titratable acidity}\left(\text{as lactic acid}\right)=\text{quantity of used NaOH }\left(\mathrm{mL}\right)/10$$

### Viscosity and Water-holding Capacity (WHC)

The values of apparent viscosity of fermented beverages were estimated via a concentric cylinder Brookfield digital rotational viscometer (Model DV-II +, Brookfield Engineering laboratories Inc., Middleboro, USA) using UL adaptor and ULA spindle over a shear rate of 12.2/s. Fermented beverages were allowed to temper at 25 °C for 10 min prior to measurement, and the readings of viscosity (centipoise, cp) were recorded. Regarding WHC, the values of WHC of beverages were assessed as described by Abd El-Fattah et al. [17].

### Microbial Population

The viable counts of *A. kunkeei*, mold and yeasts, and coliform bacteria were estimated in fermented beverages on day 1 and after 15 days of cold storage as described by APHA [18]. One milliliter of fermented beverage sample was suspended in 9 mL of sterile saline solution (NaCl 0.9%, w/v), well-vortexed and diluted serially utilizing saline solution. The viable counts (Log CFU/mL) of *A. kunkeei* culture, mold and yeasts, and coliform bacteria were enumerated on MRS agar, malt extract agar, and MacConkey agar media incubated at 30, 25, and 37 °C for 48 h, 5 days, and 24 h, respectively.

### Sensory Assessment

The sensory assessment of fermented beverages was evaluated on day 1 and after 15 days of cold storage at 5 ± 1 °C. Twenty participants from the staff members of the dairy department, Faculty of Agriculture, Cairo University, Egypt evaluated the fermented beverages samples for taste, sedimentation, appearance, odor, and overall acceptability using a 10-point hedonic scale where one indicated dislike completely and 10 indicated like completely. All participants were aware of standard sensory evaluation processes, and they had access to distilled water to clean their palates prior evaluation. Fermented beverages samples were brought out the refrigerator and were coded randomly one h prior to estimation to gain room temperature.

### Proteolytic Activity

The values of proteolytic activity were spectrophotometrically assessed by reacting generated free amino groups with *O*-phthaldialdehyde (OPA) as described by Abd El-Fattah et al. [19].

### Angiotensin Converting Enzyme-inhibitory (ACE-I) Activity

The ability of WSEs to inhibit ACE activity was evaluated spectrophotometrically according to the method of Abd El-Fattah et al. [19]. Briefly, 50 μL sample of the WSE was mixed with an equal volume of an ACE solution (0.25U). Following incubation at 37 °C for 10 min, substrate solution was added, then the mixture was incubated at 37 °C for 60 min, followed by termination of the reaction and centrifuged at 14,100 x*g* for 5 min. The absorbance was measured at 228 nm using the spectrophotometer. The control sample was prepared by replacing the WSE sample with distilled water. The inhibition (%) was calculated as follows: Inhibition (%) = (A_control_ – A_sample_)/A_control_ × 100, where A_control_ and A_sample_ are the absorbance values of the control and WSE sample, respectively.

### Antioxidant Ability

The antioxidant ability of fermented beverages was conducted spectrophotometrically using two methods including DPPH free radical scavenging and metal ions chelating as demonstrated by Abd El-Fattah et al. [20].

### Antimicrobial Activity

Fungi of *Aspergillus flavus* RCMB 002002, *Aspergillus niger* RCMB 002005, *Penicillium marneffei* RCMB 001001, *Penicillium italicum* RCMB 001018, and *Geotricum candidum* RCMB 041001, Gram-positive bacteria of *Staphylococcus aureus* ATCC 25923, and Gram-negative bacteria of *Escherichia coli* ATCC 25922 and *Salmonella typhimurium* ATCC 14028 were used to examine the antimicrobial activity of WSEs of fermented beverages using agar well diffusion technique [21, 22]. Standard antibiotics of ketoconazole (100 μg/mL) and gentamycin (4 μg/mL) were applied for differentiation as positive control samples.

### Organic acid Profile

The organic acid profile of *A. kunkeei* was analyzed in the cell-free supernatant according to Hassan et al. [23]. Twenty microliters of the supernatant were injected into an Agilent 1200 high performance liquid chromatography (HPLC) system with a Refractive Index Detector and a REFEX 8 μm 8% H Organic Acid Rezex^@^ column (Phenomenex). Sulfuric acid (5 mmol/L) was utilized as an elution fluid under conditions (flow rate = 0.6 mL/min and temperature of the column kept at 65 °C). The original standards of organic acids were performed under the same conditions. The retention time of peaks of the supernatant was compared with those of organic acid standards and quantified by determining area down the peaks.

### Anticancer Activity

The cytotoxicity ability of WSEs of fermented beverages against colon cancer Caco2 cell lines was conducted utilizing 3-(4,5-dimethylthiazol-2-yl)−2,5-diphenyltetrazolium bromide (MTT) assay [24]. Cells (1 × 10^5^ cells/mL) were implanted into 96-well plate, then the plate was incubated at 37 °C for one day, and the cell monolayer was washed twice. The wells of plate were inoculated with WSEs at various concentrations (31.25, 62.5, 125, 250, 500, and 1000 μg/mL) and were incubated (37 °C). MTT solution (20 μL, 5 mg/mL) was added to each well and shaking at 150 rpm for 5 min, then the plate was incubated (37 °C/4 h). After that, the medium was eliminated, and the formazan, a MTT metabolic substance, was solved in 200 μL of dimethyl sulfoxide. Using an ELISA reader, the optical density (OD) was read at 560 nm and subtract background at 620 nm. The OD readings should be directly related to cells amount.

### Antidiabetic Activity

The inhibition of α-amylase and α-glucosidase was utilized to determine the antidiabetic ability of fermented beverages as described by Ayyash et al. [25]. The concentrations of 1.95, 3.9, 7.81, 15.62, 31.25, 62.5, 125, 250, 500, and 1000 μg/mL were utilized as a final level of water-soluble extract of each fermented beverage for the evaluation of IC_50_. Acarbose agent was used as a positive control.

### Statistical Analysis

A randomized complete block designing and analysis of variance (ANOVA) of factorial methods were carried out utilizing Mstat-C program (Michigan State University). All parameters were assayed in three replicates, and the results were illustrated as the mean ± standard deviation. The least significant difference (LSD) test was utilized to compare among the means of parameters at the probability of ≤ 0.05. Heatmap correlation matrix plot was carried out using R statistical program (version 4.4.2).

## Results

### Chemical Composition of Fermented Beverages

The chemical composition of four fermented whey and buttermilk beverages is typical for this type of fermented products (Table 1). The slight variations in total solids among fermented beverages are acceptable in fermented dairy beverage manufacturing. The fermented buttermilk beverages with/without date pulp had the significant highest total solids, and this is due to the elevated contents of protein (4.05%), fat (0.8%), and ash (0.77%) in buttermilk compared to sweet whey (3.50% protein, 0.3% fat, and 0.56% ash) as well as Rutab date pulp contains 1.5% protein.

**Table 1:** Chemical composition (%) of fermented beverages

Beverages	Total solids	Protein	Fat	Ash
FW	11.31 ± 0.01^d^	3.21 ± 0.02^b^	0.59 ± 0.00^d^	0.60 ± 0.01^b^
FDW	11.72 ± 0.17^c^	3.28 ± 0.11^b^	0.74 ± 0.02^c^	0.62 ± 0.00^b^
FB	12.07 ± 0.21^b^	4.05 ± 0.11^a^	0.83 ± 0.00^b^	0.82 ± 0.00^a^
FDB	12.34 ± 0.01^a^	4.13 ± 0.01^a^	0.92 ± 0.01^a^	0.81 ± 0.01^a^
LSD	0.1094	0.1895	0.3460	0.1094

Means with different superscript small letters indicate significant differences among fermented beverages for each parameter. FW: fermented whey, FDW: fermented whey supplemented with date pulp, FB: fermented buttermilk, FDB: fermented buttermilk supplemented with date pulp

### Physical Characteristics of Fermented Beverages

The titratable acidity and pH values of fermented beverages on day 1 and after 15 days of cold storage are listed in Table 2. On day one of cold storage, the acidity and pH values of all fermented beverages ranged from 0.457 to 0.467% and from 5.107 to 5.25, respectively. After 15 days of storage, the acidity of all fermented beverages increased significantly, while pH values had the opposite trend.

**Table 2:** Titratable acidity, pH, Viscosity and WHC of fermented beverages

Beverages	Storage days	Acidity (%)	pH	Viscosity (cp)	WHC (%)
FW	1	0.47 ± 0.01^e^	5.25 ± 0.12^a^	18.89 ± 0.60^g^	10.00 ± 9.03^g^
	15	0.54 ± 0.05^d^	4.59 ± 0.15^b^	12.71 ± 0.00^h^	8.75 ± 0.12^h^
FDW	1	0.46 ± 0.06^ef^	5.15 ± 0.11^a^	35.79 ± 0.06^e^	33.43 ± 0.15^e^
	15	0.71 ± 0.03^b^	3.98 ± 0.17^e^	28.76 ± 0.43^f^	29.39 ± 0.55^f^
FB	1	0.46 ± 0.02^f^	5.11 ± 0.23^a^	163.10 ± 0.54^c^	45.06 ± 1.22^d^
	15	0.63 ± 0.08^c^	4.38 ± 0.21^c^	159.80 ± 0.70^d^	48.38 ± 0.27^c^
FDB	1	0.46 ± 0.05^ef^	5.11 ± 0.15^a^	349.40 ± 0.55^a^	62.91 ± 0.47^b^
	15	0.74 ± 0.04^a^	4.18 ± 0.23^d^	327.80 ± 0.74^b^	64.84 ± 0.51^a^
LSD		0.2998	0.1896	2.864	0.7573

Means with different superscript small letters indicate significant differences among fermented beverages for each parameter. WHC: water-holding capacity. FW: fermented whey, FDW: fermented whey supplemented with date pulp, FB: fermented buttermilk, FDB: fermented buttermilk supplemented with date pulp

As demonstrated in Table 2, significant variations were noted between fermented whey and fermented buttermilk beverages, and the supplementation of date pulp significantly affected the values of water-holding capacity (WHC) and viscosity on day one of cold storage. On day 15 of cold storage, the viscosity of all fermented beverages and WHC of fermented whey beverages decreased significantly, whilst the WHC of fermented buttermilk beverages increased significantly.

### The Viability of *A. Kunkeei* in Fermented Beverages

**Fig. 2:**
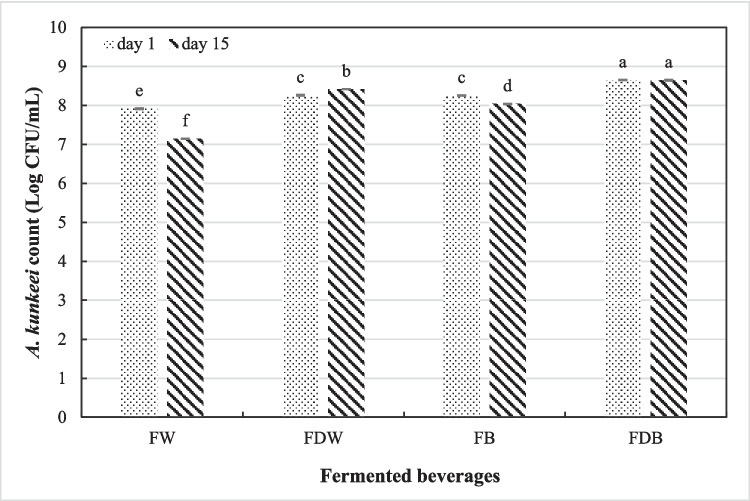
The viable count of *A. kunkeei* in fermented beverages on day 1 and after 15 days of cold storage. FW: fermented whey, FDW: fermented whey supplemented with date pulp, FB: fermented buttermilk, FDB: fermented buttermilk supplemented with date pulp

All fermented beverages were free from coliform bacteria and molds and yeasts count on day one and after 15 days of cold storage. At the beginning of cold storage, significant variations were observed among fermented beverages in the viable counts of *A. kunkeei* strain that were found between 7.91–8.65 Log CFU/mL (Fig. 2). In addition, the results showed that *A. kunkeei* strain grew well in the whey and buttermilk supplemented with Rutab date pulp, and fermented buttermilk supplemented with date pulp exhibited the highest viable count followed by fermented whey supplemented with date pulp, fermented buttermilk, and fermented whey, respectively. After 15 days of cold storage, the viable counts of *A. kunkeei* strain declined significantly to 7.14 and 8.04 Log CFU/mL in the fermented whey and buttermilk beverages, respectively. However, a significant increase was noted in the viable counts of *A. kunkeei* strain (8.41 Log CFU/mL) in the fermented whey beverage supplemented with Rutab date pulp, while a non-significant change was observed in the fermented buttermilk beverage supplemented with Rutab date pulp.

### Sensory Assessment of Fermented Beverages

**Fig. 3:**
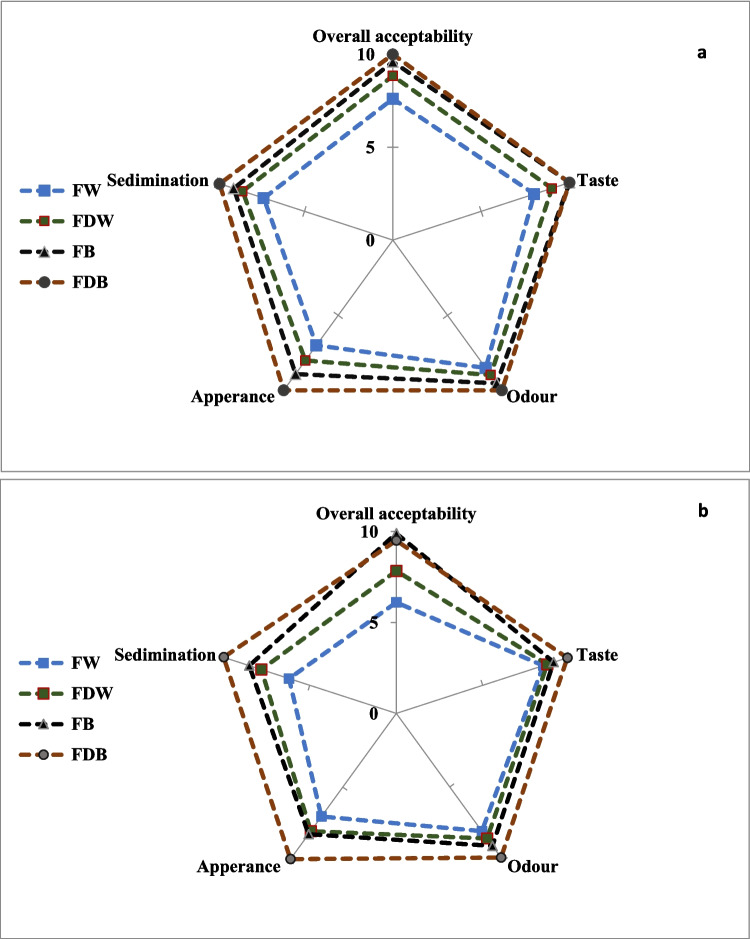
A hedonic scale for sensory attributes of fermented beverages on day 1 (**a**) and after 15 days (**b**) of cold storage.FW: fermented whey, FDW: fermented whey supplemented with date pulp, FB: fermented buttermilk, FDB: fermented buttermilk supplemented with date pulp. LSD = 0.2637

A 10-hedonic scale method was used to assess the effect of *A. kunkeei*, and Rutab date pulp on the taste, sedimentation, appearance, odor, and overall acceptability characteristics of fermented beverages on day one or after 15 days of cold storage (Fig. 3). The results demonstrated that fermented buttermilk beverages either supplemented with date pulp or without supplementation recorded the highest scores of all sensory characteristics compared to fermented whey beverages. This result indicated that buttermilk components may enhance the sensory characteristics of fermented beverages. Fermented buttermilk supplemented with date pulp exhibited significantly the greatest scores of sensory attributes compared to other fermented beverages.

### Proteolytic Activity of Fermented Beverages

**Fig. 4:**
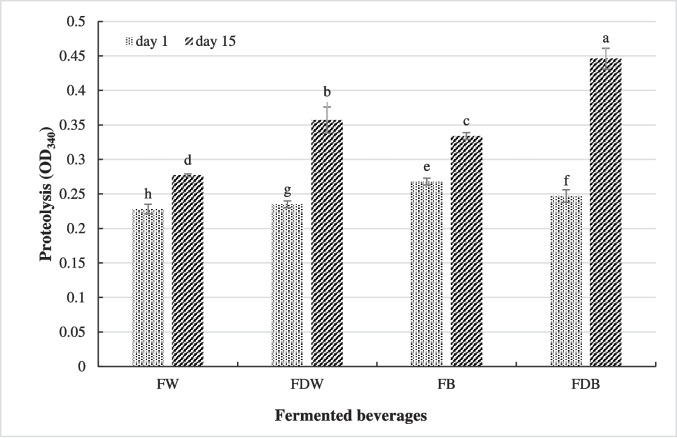
Proteolytic activity of fermented beverages on day 1 and after 15 days of cold storage. FW: fermented whey, FDW: fermented whey supplemented with date pulp, FB: fermented buttermilk, FDB: fermented buttermilk supplemented with date pulp. LSD = 0.06318

The effect of *A. kunkeei* on the proteolytic activity of fermented beverages was estimated by the level of free NH_3_ groups as illustrated in Fig. 4. OPA analysis showed that the significant greatest proteolysis occurred in the fermented buttermilk beverage (0.268) or fermented buttermilk beverage supplemented with date pulp (0.446) on day one and after 15 days of cold storage, respectively. However, the significant lowest proteolysis was observed in the fermented whey beverage on day one (0.228) or 15 th (0.277) of cold storage. Furthermore, cold storage had clearly a positive impact on the proteolytic activity of all fermented beverages, and the supplementation with date pulp exhibited a great effect on the increase of proteolysis.

### ACE-I Activity of Fermented Beverages

As presented in Table 3, the maximum ACE-I values were registered in the fermented buttermilk supplemented with Rutab date pulp on day one (42.76%) and after 15 days (66.62%) of cold storage, while the fermented whey had significantly the minimum value on day one (27.35%) or after 15 days (39.40%) of cold storage. The ACE-I values increased significantly at the rate of 30.58–35.82% with storage time in all fermented beverages. In addition, the results showed a strong correlation between proteolysis and ACE-I activity (*r* = 0.96).

**Table 3:** ACE-I, DPPH radical scavenging, and metal chelating activity (%) of fermented beverages on day 1 and after 15 days of cold storage

Beverages	Storage days	ACE-I	DPPH radical scavenging	Metal chelating
FW	1	27.35 ± 1.03^f^	32.22 ± 1.59^g^	19.96 ± 0.58^ef^
	15	39.40 ± 0.62^de^	38.14 ± 0.94^f^	20.93 ± 0.53^e^
FDW	1	36.40 ± 0.62^e^	51.26 ± 0.99^e^	28.86 ± 0.35^d^
	15	54.37 ± 0.56^b^	55.06 ± 0.30^cd^	37.96 ± 0.28^b^
FB	1	30.79 ± 1.22^f^	52.94 ± 0.64^de^	35.05 ± 0.20^bc^
	15	47.41 ± 0.64^c^	57.47 ± 0.75^c^	34.73 ± 0.41^c^
FDB	1	42.76 ± 0.47^d^	72.58 ± 0.76^b^	50.86 ± 0.57^a^
	15	66.62 ± 0.88^a^	80.64 ± 1.12^a^	53.07 ± 0.76^a^
LSD		3.983	3.836	3.208

Means with different superscript small letters indicate significant differences among fermented beverages for each parameter. FW: fermented whey, FDW: fermented whey supplemented with date pulp, FB: fermented buttermilk, FDB: fermented buttermilk supplemented with date pulp

### Antioxidant Ability of Fermented Beverages

DPPH radical scavenging capacity and metal chelating activity were utilized to estimate the antioxidant ability of fermented beverages (Table 3). All fermented beverages showed various degrees of antioxidant ability either on day one or after 15 days of cold storage. Among all beverages, the fermented buttermilk supplemented with date pulp recorded the significant strongest antioxidant ability with two methods, whilst the fermented whey had an opposite trend. The storage period had a positive effect on the DPPH radical scavenging capacity of all fermented beverages.

### Antagonistic Activity of Fermented Beverages

**Fig. 5:**
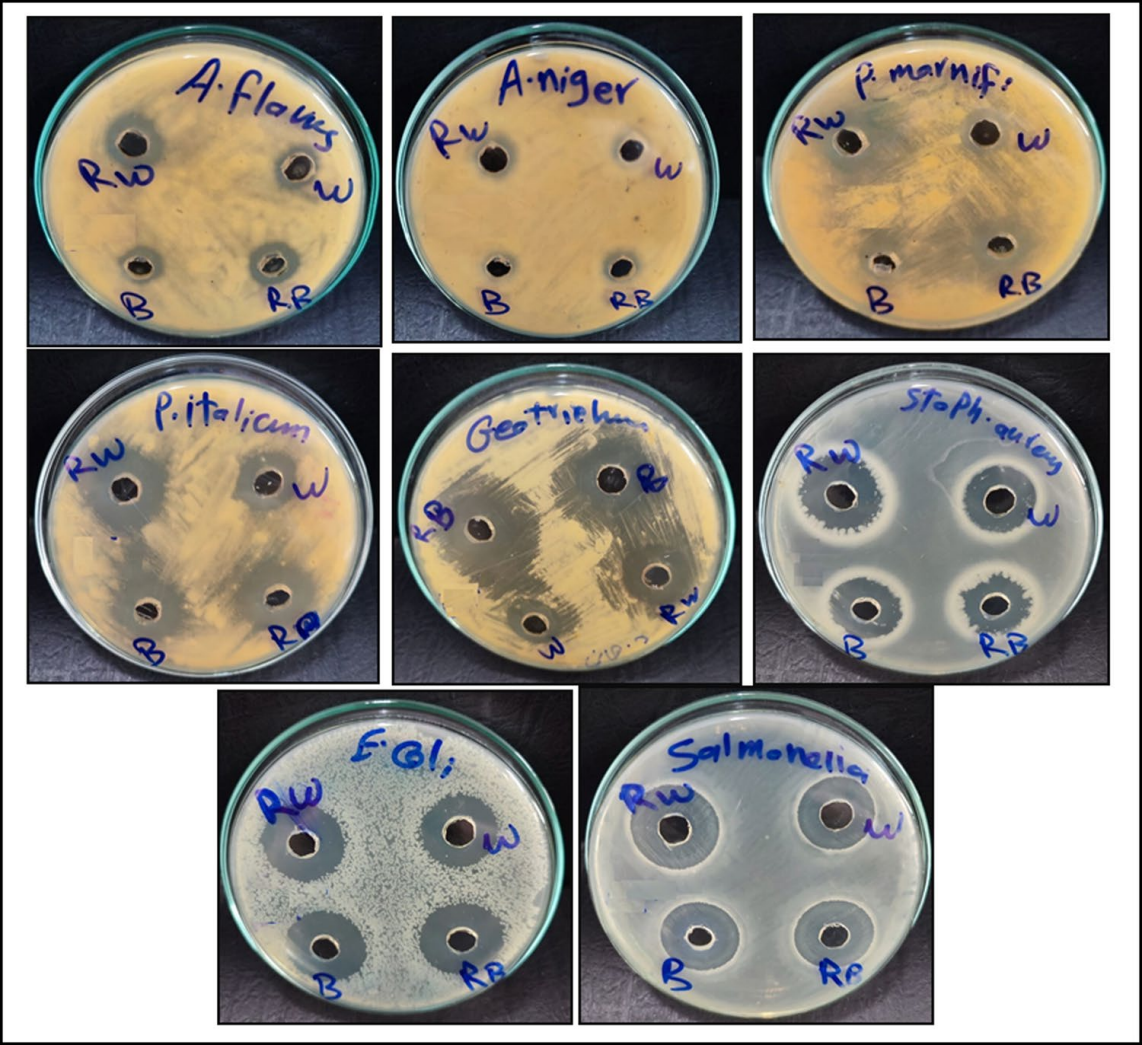
Inhibitory effect of fermented beverages against *A. flavus*, *A. niger, P. marneffei, P. italicum, Geotricumcandidum, Staph. aureus, E. coil, and Sal. typhmurium*. W: fermented whey beverage, RW: fermented whey beverage with Rutab date pulp, B: fermented buttermilk beverage, RB: fermented buttermilk beverage with Rutab date pulp

The ability of WSE of fermented beverages to inhibit the tested G^+^ and G^−^ bacteria and fungi is shown in Table 4 and Fig. 5. It was observed that all fermented beverages appeared considerably the antibacterial and antifungal with various levels. The diameters of inhibition zone against the tested fungi ranged from 9–27 mm, while it was 16–26 against the tested bacteria. Fermented buttermilk supplemented with date pulp recorded the highest inhibition zone diameters against all microorganisms except *Penicillium marneffei.*

**Table 4:** The antimicrobial activity of fermented beverages after 15 days of cold storage

**Tested microorganisms**	Fermented beverages				Control
	FW	FDW	FB	FDB	
**Fungi**	Zones of inhibition (mm)				Ketoconazole
*Aspergillus flavus* RCMB 002002	10.00 ± 0.05^e^	14.00 ± 0.00^e^	13.00 ± 0.01^e^	15.00 ± 0.02^f^	15.00 ± 0.00
*Aspergillus niger* RCMB 002005	9.00 ± 0.15^f^	10.00 ± 0.01^f^	9.00 ± 0.00^f^	11.00 ± 0.01^g^	16.00 ± 0.01
*Penicillium marneffei* RCMB 001001	12.00 ± 0.17^d^	18.00 ± 0.12^d^	13.00 ± 0.01^e^	17.00 ± 0.05^e^	17.00 ± 0.03
*Penicillium italicum* RCMB 001018	16.00 ± 0.05^c^	23.00 ± 0.07^a^	20.00 ± 0.57^d^	26.00 ± 0.57^b^	18.00 ± 0.05
*Geotricum candidum* RCMB 041001	17.0 ± 0.01^b^	20.00 ± 0.02^c^	21.00 ± 0.55^c^	27.00 ± 0.57^a^	26.00 ± 0.10
**Gram positive bacteria**	Zones of inhibition (mm)				Gentamycin
*Staphylococcus aureus* ATCC 25923	17.00 ± 0.05^b^	20.00 ± 0.00^c^	21.00 ± 0.00^c^	20.00 ± 0.05^d^	24.00 ± 0.10
**Gram negative bacteria**	Zones of inhibition (mm)				Gentamycin
*Escherichia coli* ATCC 25922	16.00 ± 0.02^c^	23.00 ± 0.51^a^	24.00 ± 0.57^a^	26.00 ± 0.57^b^	30.00 ± 0.05
*Salmonella typhimurium* ATCC 14028	19.00 ± 0.01^a^	22.00 ± 0.03^b^	22.00 ± 0.00^b^	23.00 ± 0.00^c^	17.00 ± 0.00

The inhibition zone greater or equal to 6 mm was selected. Data are means ± SD (n = 3)

Means with different superscript small letters in the same row indicate significant differences

FW: fermented whey, FDW: fermented whey supplemented with date pulp, FB: fermented buttermilk, FDB: fermented buttermilk supplemented with date pulp

RCMB: Regional Center for Mycology and Biotechnology, Faculty of Pharmacy, Al-Azhar Univ., Cairo, Egypt

### Anticancer Activity of Fermented Beverages

In vitro evaluation has been carried out to determine the cytotoxicity of fermented beverages using *A. kunkeei* at the end of cold storage against the viability of human cancer cell lines. The cell proliferation method as cytotoxic impact of the WSE of fermented beverages on Caco2 cells with different levels was conducted by 3-(4,5 dimethyl thiazal-2-yl)−2,5-diphenyl tetrazolium bromide (MTT). Anticancer activity of different fermented beverages was expressed as IC_50_, which is recognized as the inhibitory level that results in 50% deactivation of the cancer cell viability (Table 5). The results illustrated that the cytotoxicity impact of each fermented beverage on cell lines enhanced by increasing studied level. The greatest cytotoxicity against Caco2 cell lines was observed for the fermented buttermilk beverage with IC_50_ value of 81.22 μg/mL, followed by the fermented buttermilk beverages supplemented with date pulp (IC_50_ 86.89 μg/mL). On the other hand, the fermented whey beverage supplemented with date pulp exhibited the lowest cytotoxicity effect with IC_50_ value of 311.90 μg/mL.

**Table 5:** IC_50_ values (μg/mL) of anticancer activity and α-amylase and α-glucosidase inhibition by fermented beverages after 15 days of cold storage

Beverages	Anticancer	Antidiabetic activity	
		α-amylase	α-glucosidase
FW	97.19 ± 0.53^b^	8.50 ± 0.12^d^	6.87 ± 0.15^d^
FDW	311.90 ± 1.40^a^	10.42 ± 0.13^c^	8.98 ± 0.21^c^
FB	81.22 ± 0.33^d^	16.64 ± 0.25^a^	22.09 ± 0.15^a^
FDB	86.89 ± 0.72^c^	10.83 ± 0.15^b^	10.49 ± 0.20^b^
Acarbose	–	5.73 ± 0.22^e^	6.52 ± 0.15^e^
LSD	1.856	0.3036	0.2977

Acarbose was used as a positive control for the inhibition of α-amylase and α-glucosidase

Means with different superscript small letters indicate significant differences among fermented beverages for each parameter. FW: fermented whey, FDW: fermented whey supplemented with date pulp, FB: fermented buttermilk, FDB: fermented buttermilk supplemented with date pulp

### Antidiabetic Activity of Fermented Beverages

The ability of fermented beverages to inhibit α-amylase or α-glucosidase enzymes as an indication to their antidiabetic activity were assessed at the end of cold storage (Table 5). The IC_50_ values of α-amylase or α-glucosidase inhibitory ability of fermented beverages followed the order of acarbose (positive control) > fermented whey beverage > fermented whey beverage supplemented with date pulp > fermented buttermilk beverage supplemented with date pulp > fermented buttermilk beverage. Also, our data showed that fermented whey beverage inhibited significantly α-amylase or α-glucosidase enzymes, and its efficiency was comparable to the acarbose (standard agent) somewhat.

## Discussion

Nowadays, consumers will prefer high-quality products that are fortified with functional natural constituents and do not contain artificial additives or preservatives. In this regard, novel fermented whey- and buttermilk-based beverages using *Apilactobacillus kunkeei* was developed. The highest total solid of fermented buttermilk with/without date pulp was due to the elevated contents of protein, fat, and ash in butter milk compared to sweet whey. Our results are like those reported by Gebreselassie et al. [26]. Additionally, the fermented whey or buttermilk beverages supplemented with date pulp had significantly the highest acidity (lowest pH values) compared to other fermented beverages, and this may be due to the great populations of *Apilactobacillus* counts. Development of acidity during fermentation or storage period is closely associated with the potential metabolism of *A. kunkeei*, when utilizing carbohydrates particularly fructose of date pulp and generating organic acids. Santos et al. [27] found similar behavior in goat whey fermented with probiotic *Lactobacillus* strains. High WHC of the fermented butter milk beverage compared to fermented whey may be attributed to the constitutes of buttermilk, especially phospholipids and proteins that possess great emulsifying and water-retention ability. In this respect, Romeih et al. [28] declared that phospholipids exhibited great WHC because of their amphiphilic characteristics. Furthermore, the dietary fibers of Rutab date pulp (2.4%) have a great impact on the ability to retain water, gel formation, and emulsifying properties [29]. After 15 days of storage, the WHC of fermented buttermilk beverages increased and this result may be explained by the greater hydration of buttermilk and the interaction between residual proteins and milk fat globule membrane compounds that enhances the viscosity and WHC during storage of fermented milk products [30]. The reduction in WHC of fermented whey beverage may be due to the weak bonds between whey proteins and H_2_O molecules and the decline in pH at the end of storage [31].

Health promoting benefits of probiotic bacteria depend on the viable counts of microorganism, and the counts of live probiotic bacteria should not be less than 6 Log CFU/g to obtain their promising health effects. High viable count of *A. kunkeei* in the fermented buttermilk supplemented with date pulp was due to a symbiotic relationship between Rutab date pulp and the growth of *A. kunkeei* where this species is considered as a candidate of fructophilic-LAB [32] and Rutab date pulp is rich in fructose (23.5%). Moe et al. [33] and Martinovic et al. [34] stated that butter milk consisted of low-molecular components such as peptides, amino acids, and milk fat globule membrane (MFGM) components, which act as growth factors for *Lactobacillus* species. Additionally, some studies have revealed the relationship between the beneficial effects of fruit pulp components and the viability of probiotic bacteria during storage of fermented beverages [35, 36]. The decline in the viable counts after 15 days of storage may be attributed to the accumulation of *A. kunkeei* metabolites including organic acids that affect the growth of LAB [37]. Generally, the viable counts of *A. kunkeei* in all fermented beverages confronted the minimum standards recommended for probiotic bacteria to reinforce health effects in the gut, surviving higher than 6 Log CFU/mL [38]. The obtained results also illustrated that no growth of either yeast, molds and coliform in all fermented beverages, this could be attributed to the hygienic conditions during manufacturing and the inhibiting effect of *A. kunkeei* as they produce a range of antimicrobial compounds, which could inhibit numerous G^+^ and G^−^ bacteria and other microbes. Sensorially, the fermented buttermilk supplemented with date pulp exhibited the greatest scores of sensory attributes. This result may be due to the presence of Lacta 533 stabilizer that contributes to the stability of fermented beverages and the sweetness of Rutab date pulp that enhances the taste of fermented beverages. In this regard, M’hir et al. [39] reported that the addition of date syrup enhanced the sensory acceptability of fermented whey beverage. Alhamdan et al. [40] found that the fortification of fermented milk (laban) drink with date syrup at a level of 12.5% improved the organoleptic properties. Costa et al. [41] declared that the fortification of fermented dairy beverages with stabilizers or thickeners had a great impact on their sensory acceptability. OPA analysis showed that the significant greatest proteolysis occurred in the fermented buttermilk beverage with/without date pulp and the supplementation with date pulp exhibited a great effect on the increase of proteolysis and this may be due to its effect on the populations of *A. kunkeei* as mentioned above. The decline of proteolysis in the fermented whey beverages may be dependent on the bacterial strain where Pescuma et al. [42] found that *L. casei, L. bulgaricus,* and *L. acidophilus* showed more proteolytic activity than *L. paracasei,* and *S. thermophilus* during cheese whey fermentation. Also, they mentioned that whey proteins, including α-lactalbumin and β-lactoglobulin, appeared high resistance to the action of proteolytic enzymes of LAB strains, particularly β-lactoglobulin. As shown in this study, *A. kunkeei* had a high proteolytic activity that increased during storage of fermented beverages, and this activity attributed to the presence of proteinases and peptidases, which hydrolyze milk proteins to various large and small peptides and amino acids. Alwohaibi et al. [15] reported that sweet buttermilk was a better medium than sweet whey for the growth of yogurt starter or *L. paracasei* strain and their proteolytic activity.

In accordance with the previous report by Li et al. [43], *A. kunkeei* may be possessed proteolytic system able to hydrolyze milk proteins into peptides containing Pro, Trp, Tyr, or Phe at their C-terminus and branched aliphatic amino acids at their N-terminal, conferring their ACE-I activity. In this concern, Abd El-Fattah et al. [19] found that 14 different *Lactobacillus* strains able to inhibit ACE with various degrees in skim milk. Pereira et al. [44] produced fermented goat whey beverage using *S. thermophilus* TA-40 and probiotic *L. casei* BGP93, as an adjunct culture, with twofold greater ACE-I activity than that of noted for the control beverage with *S. thermophilus* only. Similar results were obtained by Magouz et al. [45] who identified 33 peptides with ACE-I ability in fermented buttermilk with *L. lactis* subsp. *cremoris*, *L. lactis* subsp. *lactis*, *Leuc. mesenteorides* subsp. *cremoris* and *L. lactis* subsp. *lactis* biovar *diacetylactis* and they reported that these peptides were generated from αs1 and αs2-casein, β-casein, and β-lactoglobulin. Abd El-Fattah et al. [19] and Mazorra-Manzano et al. [46] found similar results concerning the positive correlation between ACE-I and the degree of proteolysis. Antioxidant activity was measured by the ability of WSE of fermented beverages to scavenge the free radicals or chelate metals. The results suggested that *A. kunkeei* generated peptides with radical scavenging and metal chelating activities. Magouz et al. [45] identified ten peptides with antioxidant ability in fermented buttermilk with *L. lactis* subsp. *cremoris*, *L. lactis* subsp. *lactis*, *Leuc. mesenteorides* subsp. *cremoris* and *L. lactis* subsp. *lactis* biovar *diacetylactis* and they mentioned that the most efficient fragment was privileged in these peptides released from the prevailing MFGM proteins particularly butyrophilin. Stobiecka et al. [47] reported that buttermilk composes worthy amounts of MFGM proteins that participate in the antioxidant capacity of fermented beverages. Furthermore, Hu et al. [49] and Wang et al. [11] observed that polar lipids of buttermilk play a very important role in the antioxidant ability; protecting the gangliosides against reactive O_2_ species. Phospholipids, containing long polyunsaturated fatty acids, are known as cation-binding ability [50]. Otherwise, the generated antioxidant peptides by *A. kunkeei* may act as electron donors because of containing aromatic and hydrophobic amino acids that interact with free radicals to make them more stable [51]. Regarding date pulp, several studies have shown that different date varieties are a considerable source of antioxidant compounds including phenolics, flavonoids, and tannins [52, 53]. Sharma et al. [54] found that the fermentation of buttermilk with *Pediococcus acidilactici* BD16 increased the DPPH radical scavenging activity from 27.2 to 67.3% compared to unfermented buttermilk. Biadała and Adzahan [55] fermented goat whey with various *Lactobacillus* strains, and they found that the fermentation with *L. acidophilus* exhibited the greatest antioxidant activity (63.43 trolox eq./100 mL) compared to other strains.

**Fig. 6:**
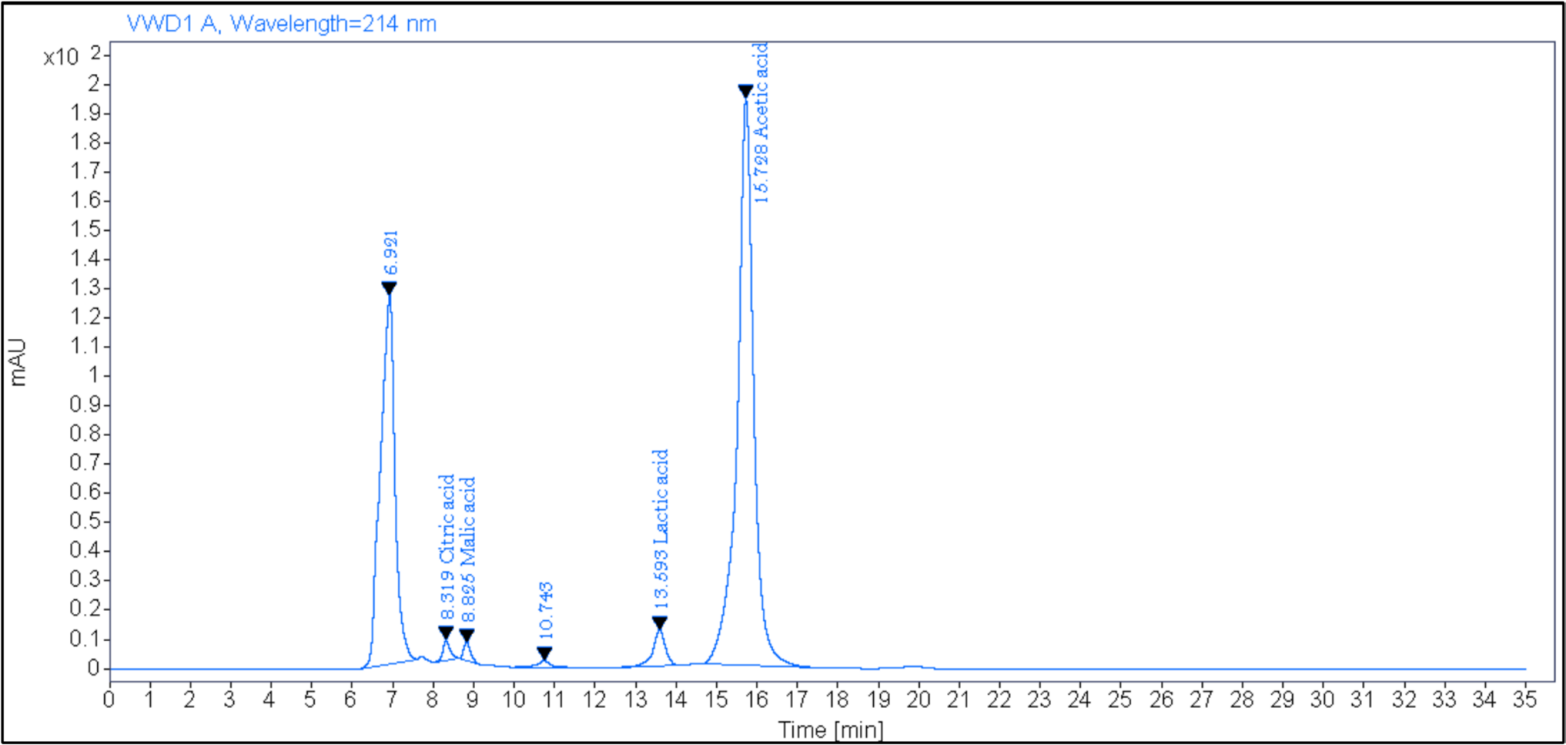
HPLC chromatogram of organic acid profile of *A. kunkeei* EABW06

Several mechanisms have been proposed to explain the antibacterial action of LAB including organic acids, hydrogen peroxide, and bacteriocins [56]. Our results indicated that *A. kunkeei* can be used to inhibit strongly various species of fungi and pathogenic bacteria such as *Staph. aureus* and *Sal. typhimurium* and that may be attributed to its production of some organic acid including citric acid (207.34 mg/L), malic acid (92.78 mg/L), lactic acid (1158.83 mg/L) and acetic acid (1974.28 mg/L) as demonstrated in Fig. 6. Also, these results may be due to generate antimicrobial peptides via whey or buttermilk proteins hydrolysis by proteolytic enzymes of *A. kunkeei*. Ounine and Attarassi [57] assessed the effect of lactic, citric, and acetic acids on the inhibition of *E. coli*, and they found that citric acid showed the greatest efficiency against *E. coli* by 6.3 Log decrease. Scott et al. [58] reported that a mixture of lactic and citric acids decreased *Salmonella* counts by Log 1.3–2.3 CFU/mL. In another study, Eswaranandam et al. [59] succeeded in reducing the viable counts of *E. coli* O157:H7, *L. monocytogenes*, and *S. gaminara* from Log 8.9, 8.3, and 9.0 to Log 6.8, 5.5, and 3.0 CFU/mL, respectively after their treatment with 2.6% malic acid. Regarding antimicrobial peptides, Magouz et al. [45] identified 11 peptides exhibiting the antimicrobial ability in fermented buttermilk and they reported that casecidin 17 (β-casein 193-209f) was one of these peptides, which had minimum inhibitory concentrations about 0.5 and 0.4 mg/mL against *E. coli* DH5a and *E. coli* DPC6053, respectively. Izzo et al. [60] stated that the goat whey fermented by *L. plantarum* has exhibited good antifungal ability against 28 species of *Aspergillus* and *Fusarium* genus and they identified phenyl lactic acid in the fermented whey at a level of 0.34–1.21 mg/L.

Exploring novel chemotherapy is a challenge, which requires great costs, time, and continuous scientific research; prompting the researchers to develop novel compounds from natural sources that could have anticancer activity [61]. Thence, In vitro evaluation has been carried out to determine the cytotoxicity of fermented beverages using *A. kunkeei* at the end of cold storage against the viability of human cancer cell lines. The anticancer activity of fermented butter milk beverage may be attributed to buttermilk containing various compounds that have anticancer activities where Kuchta-Noctoret al. [62] reported that sphingomyelin and lactosylceramide inhibited the growth of SW480 colon cancer cells In vitro. Hirayama and Rafter [63] and Kumar et al. [64] stated that LAB particularly probiotic bacteria can inhibit colon cancer using various mechanisms including binding and breaking carcinogenic agents, improving the host immune system, changing physicochemical circumstances in the colon, and generating antimutagenic molecules. Regarding milk anticancer peptides, Kandyliari et al. [65] and Pessione and Cirrincione [66] proposed several mechanisms for these peptides including the competition between the milk peptides and cancer growth factors for cancer cell-membrane receptors and possessing specific cytotoxicity on cancer cells: inducing apoptosis. Moreover, Hsieh [67] and Iigo et al. [68] reported that ingestion of bovine lactoferrin inhibits carcinogenesis in the colon. Further, whey proteins had anticancer properties and that may be because of their capability to enhance cellular concentrations of glutathione, acting as an antioxidant, and improve the responses of hormonal and cell-mediated immune. Rosa et al. [69] evaluated the antiproliferative effect of probiotic whey beverages fermented with *L. acidophilus* La-05, *L. acidophilus* La-03, *L. casei*−01, or *B. animalis* Bb-12 on human prostate cancer cell lines (DU-145 and PC-3), and they found all whey beverages exhibited cytotoxicity actions against both cell lines, and the whey beverage fermented with *L. casei*−01 was the best candidate against prostate cancer cells.

Alpha-amylase enzyme metabolizes dietary carbohydrates into simple monosaccharides through the digestive tract, and α-glucosidase converts these monosaccharides into glucose to enter the bloodstream. Hence, the suppression or inhibition of these enzymes can retard glucose absorption and thus decrease blood sugar concentrations [70]. The results showed a high antidiabetic activity in the fermented whey beverages. In this respect, Rosa et al. [71] found that milk whey beverages fermented with *L. acidophilus* La-03, *L. acidophilus* La-05, *Bifidobacterium* Bb-12 or *L. casei*−01 displayed α-amylase and α-glucosidase inhibition, particularly after 15 days of storage. They attributed these results to the ability of probiotic bacteria to hydrolysis of whey proteins into biopeptides that can inhibit these enzymes. Shirkhan et al. [72] fermented UHT-milk with ten strains of *Lactobacillus* separately and investigated the antidiabetic activity of fermented milk via the determination of α-amylase or α-glucosidase inhibition. They found that milk fermented with *L. helveticus* PTCC 1930 or *L. paracasei* subsp. *paracasei* PTCC 1945 had the greatest enzymes inhibitory abilities after 48 h of fermentation. Also, they declared that antidiabetic ability was associated with the type of strain. Furthermore, they attributed the variation among *Lactobacillus* strains in the inhibition of these enzymes to the type and quality of released peptides, and the interaction between peptides and the active site of enzyme. Raveschot et al. [73] reported that LAB strains generate various metabolites during fermentation such as vitamins, lactate, and exopolysaccharides; having the ability to inhibit α-glucosidase and α-amylase enzymes. The mechanism of deactivate of these enzymes may be related to the different capacities of peptides. Yan et al. [74] reviewed that α-amylase inhibitory peptides have been recognized to impede the catalytic site of enzyme or impede the substrate and catalytic-binding sites. Castañeda-Pérez et al. [75] and Famuwagun et al. [76] mentioned that short-chain or small peptides had the greatest inhibitory ability against α-glucosidase and α-amylase enzymes, and they attributed that to the liberation of electrons or the presence of some amino acid residues, which bind to the active or catalytic sites of enzyme and impede enzyme functions.

**Fig. 7:**
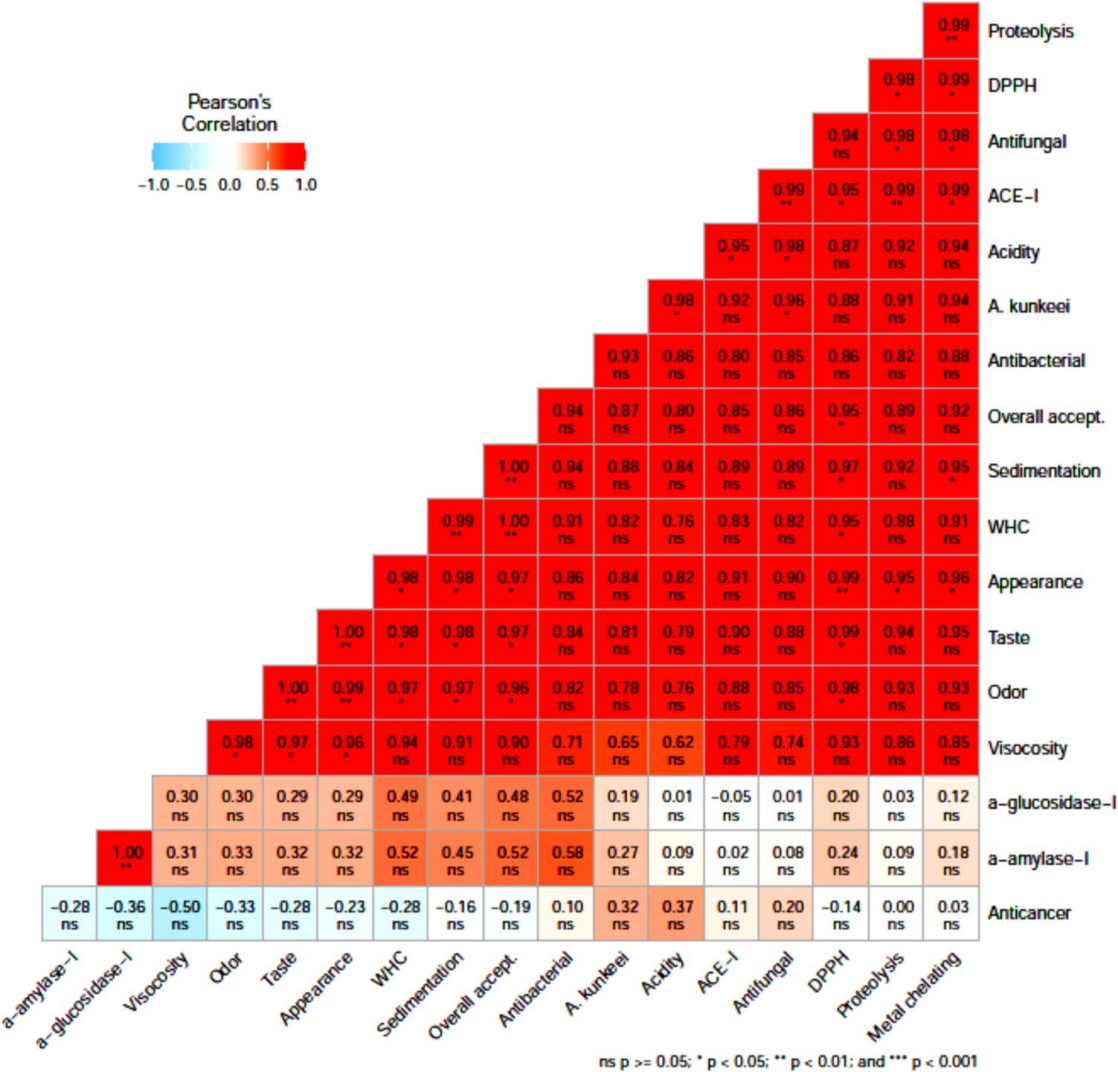
Heatmap plot performing the correlations matrix among the studied properties of different fermented dairy beverages. Positive values point out positive relationships among two properties (variables). The significance (*) indicates the effect of fermented dairy beverages (treatments) on the relationships among variables

Based on the parameters that stating physical, sensory, and bioactive characteristics of different fermented beverages, the interrelationships among the studied variables as well as the effect of *A. kunkeei* and Rutab date (fermented beverage treatments) on these relationships were performed as shown in heat map data visualization plot to deeply assess the overall interrelationships (Fig. 7). Color points out the correlation among various variables, in which red color points out high relationships or correlation, whilst light blue indicates a weak or negative correlation. It was observed that α-amylase or α-glucosidase inhibition and anticancer activity appeared a weak or negative correlation with other variables. These results may indicate that excessive proteolysis may break down anticancer or antidiabetic peptides and alter in the net charge on peptides, resulting in their efficiency [77]. On the other hand, the heat map plot demonstrates that different fermented dairy beverages (treatments) exhibited a significant effect on the interrelationship between proteolysis and ACE-I, DPPH radical scavenging, or antifungal activity. These findings showed that protein hydrolysis by probiotic *A. kunkeei* strain led to produce peptides with different molecular weights, resulting in diverse biological functions [78]. In addition, there was a significant impact of different treatments on the interrelationship between physical properties (viscosity and WHC) and sensory attributes. A significant influence was noted for using *A. kunkeei* as a probiotic candidate on the antifungal activity of fermented beverages. This result proved that *A. kunkeei* might be produced various metabolites affecting negatively the growth of fungi besides antifungal peptides.

## Conclusion

This work provided an approach to valorize cheese whey and buttermilk to promote their sustainability as food by fermentation with a probiotic *A. kunkeei* EABW06 strain. The results suggested that whey or buttermilk is considered a good medium for the growth of *A. kunkeei*, particularly in the presence of Rutab date pulp. Fermented dairy byproducts showed a good food carrier for delivering probiotic *A. kunkeei* strain, but concerning sensory assessment, fermented buttermilk beverage supplemented with date pulp recorded the best acceptability. High proteolytic, ACE-I, and antioxidant activities were observed in the fermented buttermilk beverages. Proteolysis, ACE-I activity, DPPH radical scavenging ability of all fermented beverages improved after 15 days of cold storage. In addition, probiotic fermented buttermilk beverages presented a nutraceutical strategy for preventing colon cancer. On the other hand, the current study proposed α-amylase and α-glucosidase inhibition as potential path of fermented whey beverage for reducing blood glucose in diabetic patients. Depending on the good activity of fermented beverages against the growth of tested fungi and bacteria, this work suggested the potential use of *A. kunkeei* as a one of novel bio-preservatives of natural source to apply in food products for enhancing their shelf life. Overall, our findings suggested the ability of dairy byproducts valorization via their fermentation with probiotic bacteria to assure their efficiency utilization and maximize their functional or health effects.

## Data Availability

No datasets were generated or analysed during the current study.
